# Gut microbiota causally affects ulcerative colitis by potential mediation of plasma metabolites: A Mendelian randomization study

**DOI:** 10.1097/MD.0000000000042791

**Published:** 2025-06-27

**Authors:** Shidong Zhao, Xianjing Zheng, Changjiang Yang, Weisong Shen, Zhanlong Shen

**Affiliations:** a Department of Gastroenterological Surgery, Laboratory of Surgical Oncology, Beijing Key Laboratory of Colorectal Cancer Diagnosis and Treatment Research, Peking University People’s Hospital, Beijing, PR China; b Department of Pathology, Third Hospital, School of Basic Medical Sciences, Peking University Health Science Center, Beijing, PR China.

**Keywords:** causal relationship, gut microbiota, Mendelian randomization (MR), plasma metabolites, ulcerative colitis (UC)

## Abstract

Ulcerative colitis (UC) is a chronic inflammatory bowel disease with a multifactorial etiology, including genetic, immunological, and environmental factors, as well as alterations in the gut microbiome and plasma metabolites. The interplay between these factors is complex and not fully elucidated, particularly regarding the potential mediation of metabolites in the relationship between gut microbiota and UC. We performed a Mendelian randomization (MR) study to investigate the causal associations between gut microbiota, plasma metabolites, and UC. The study utilized a two-sample MR approach to discern causal relationships among these factors. Genetic variants from genome-wide association studies served as instrumental variables in the MR analyses, conducted using the “TwoSampleMR” package in R software. We adhered to the fundamental assumptions of MR analyses, ensuring the validity of our causal inferences. Additionally, we incorporated a mediation analysis to assess the potential mediating role of plasma metabolites in the relationship between gut microbiota and UC. Our current study found the substantial relationship between certain gut microbial taxa and the development of UC. Indeed, we have identified 6 microbial taxa, including Genus *Dorea*, Phylum Proteobacteria, Species *Streptococcus parasanguinis*, Species *Ruminococcus obeum*, Species *Roseburia intestinalis*, and Order Lactobacillales, which were found to be causally related to UC. Seventy-three metabolites and metabolite ratios of were also causally associated with UC, and a mediation analysis revealed that metabolites such as stearoylcarnitine, 3-hydroxyoctanoylcarnitine, 1-arachidonoyl-GPE (20:4n6), 3-(3-hydroxyphenyl)propionate sulfate, and thioproline mediated the effects of gut microbiota on UC and hence might play roles in disease pathogenesis. This microbiota–UC-specific MR study provides evidence for causal associations between specific gut microbiota and UC, potentially mediated through plasma metabolites. The findings give new perspectives on the causal nexus of the gut microbiota and plasma metabolites with UC, highlighting potential intervention targets for the disease. These findings call for confirmation in further research, together with investigation of the underlying mechanisms.

## 1. Introduction

Ulcerative colitis (UC) is a chronic inflammatory bowel disease (IBD) characterized by inflammation of the mucous membrane of the colon and rectum. Common symptoms usually include bloody diarrhea, with attendant abdominal pains and a frequent urge to the bowel movement. The course of the condition often consists of relapses combined with periods of remission. The causes are multifactorial, involving genetic predispositions, immune dysregulation, environmental factors, and important changes in the gut microbiome. UC represents a complex interplay between genetic susceptibility and environmental triggers. While the exact nature of this process remains poorly understood, it is well established that the disease is more common amongst those with a family history of IBD and with specific human leukocyte antigen gene variants. These include dietary factors, stress, and some medications, which may precipitate or worsen UC. The goals for the treatment of UC are induction and maintenance of remission, reduction of the frequency and severity of relapses, and improving the quality of life. This could include medication to reduce inflammation, suppress the immune system, and biological treatments. Surgery also sometimes becomes indispensable, especially in cases of severe disease that is unresponsive to medical treatment.^[1]^

Gut microbiota is an ecological unit within the human body, capable of expressing complex physiology, modulating a snail of physiological functions which include the immune response, essential for maintaining host health. Where the representation of microbiota is balanced, it helps in maintaining the integrity of the gut lining and prevents the invasion by disease-causing pathogens. In contrast, disturbance or imbalance-dysbiosis-in this microbial community may lead to a break in these important processes and can result in several diseases, including IBD. Dysbiosis is typically characterized by the loss of beneficial microorganisms, reduced microbial diversity, and the appearance of pathogenic bacteria. Indeed, regarding diseases such as UC and Crohn disease, it has been documented that dysbiosis is characterized by decreased levels of protective bacteria, including *Faecalibacterium prausnitzii*, but increased levels of microbes with pro-inflammatory activity. Such changes in gut microbiota are thought to disrupt the intestinal barrier, thus precipitating inappropriate immune responses, thereby contributing to chronic inflammation characterized in IBD. Treatment approaches towards gut microbiome restoration include dietary interventions, probiotics, prebiotics, and fecal microbiota transplantation (FMT).^[2,3]^ Approaches along these lines are necessary for the reinstitution of a balanced gut microbiota, hence countering dysbiosis, thereby alleviating symptoms brought about by IBD, as well as other gut-related ailments. Of these, FMT has emerged as a promising treatment option for recurrent *Clostridioides difficile* infection and may be of benefit in IBD.^[4]^ However, further studies on optimal strains and dosing and duration of treatment are necessary to establish the role of probiotics in UC.

Metabolism in UC is correlated in an extraordinary and multi-dimensional manner with the complex interplay shaping the gut microbiota, through modulation of host immunity, energy metabolism, and inflammatory responses.^[5]^ The gut microbiome forms characteristic changes in patients with UC, with their metabolic functions affecting the production of short-chain fatty acids (SCFAs), an important player in the maintenance of intestinal barrier integrity and immune regulation.^[6]^ The imbalance of the microbiome and a reduction of beneficial bacteria, such as *Bifidobacterium* and *Lactobacillus*, while potentially damaging bacteria increase, may generate a reduction of SCFA, compromising the integrity of the intestinal barrier and favoring inflammation. Moreover, in UC, immune derangement, and metabolic pathway modifications could affect immune cells functional activity and the inflammatory response.^[7]^ In a general view, the metabolic pathways in UC are related almost to all aspects of the disease, and the changes in metabolisms may interact with each other in the pathogenesis of UC.

We used Mendelian randomization (MR), an instrumental variable (IV) analysis with single nucleotide polymorphisms (SNPs) as instruments to assess the causality between 2 traits. MR has 2 additional advantages: it can remove the bias due to confounding and solve the problem due to reverse causality.^[8]^ Therefore, we performed a MR analysis to test the causality between gut microbiota, plasma metabolome, and UC.

Our research investigated the association between gut microbiota and UC, then explored whether metabolic products might act as a mediator to perform a bidirectional two-step MR analysis.

## 2. Materials and methods

### 2.1. Study design

**Figure 1.**
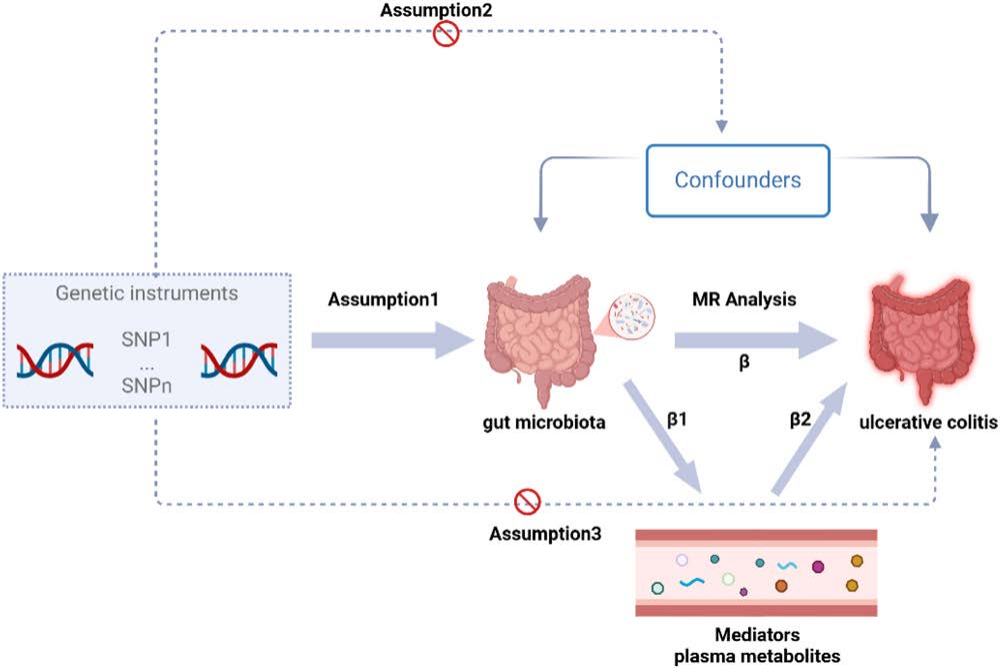
The study design. A two-step MR study of gut microbiota on ulcerative colitis mediated by plasma metabolites. MR = Mendelian randomization.

The research utilized a two-sample MR analysis to identify causal links between gut microbiota, plasma metabolites, and UC. This analysis treated gut microbiota and plasma metabolites as exposures, while UC was considered the outcome. The study design is illustrated in Figure 1. The study is divided into 2 stages. In the first stage, we used a two-sample MR method, considering gut microbiota and plasma metabolites exposure factors and UC as the outcome. We aimed to identify gut microbiota and plasma metabolites highly associated with UC risk. Additionally, we conducted a reverse MR analysis using SNPs associated with UC as IVs to explore potential causal relationships between UC and the gut microbiota significantly associated with UC. Gut microbiota that were positive in the reverse MR analysis were excluded from further analysis. In the second stage, after identifying gut microbiota and plasma metabolites related to UC risk, we further assessed the causal effects of gut microbiota on these plasma metabolites and calculated the proportion of the effect of gut microbiota on UC mediated by each plasma metabolite. Through this rigorous approach, we aim to elucidate the causal relationship between gut microbiota and UC and explore the potential role of plasma metabolites in this process. Genetic variants from the genome-wide association study (GWAS) database were utilized as IVs in two-sample MR analyses conducted using the “TwoSampleMR (0.6.1)” package in R software (4.4.0). The study adhered to 3 fundamental assumptions for MR analyses: (1) a significant correlation between IVs and exposure factors, (2) no correlation between IVs and any confounding factors associated with both exposure and outcome and (3) the exclusive influence of IVs on outcomes via exposures.^[8]^ All datasets utilized in this study are publicly available, and ethical approval was obtained from the corresponding institutions for each GWAS involved. Our study was performed following the STrengthening the Reporting of OBservational studies in Epidemiology – Mendelian Randomization (STROBE-MR) Guidelines.^[9]^

### 2.2. Data sources

GWAS data sources are summarized in Table S1, Supplemental Digital Content, https://links.lww.com/MD/P157. All procedures followed were by the ethical standards of the responsible committee on human experimentation (institutional and national) and with the Declaration of Helsinki of 1975, as revised in 2008. All studies included in the cited GWAS had been approved by a relevant review board. All participants provided informed consent.

GWAS data for gut microbiota were obtained from the Dutch Microbiome Project. Lopera-Maya et al, identified 207 taxa and 205 pathways, delineating microbial composition and function,^[10]^ plasma metabolites data were derived from the comprehensive metabolite statistics including 1091 metabolites and 309 metabolite ratios in the NHGRI-EBI GWAS Catalog. The dataset was derived from 8299 individuals from The Canadian Longitudinal Study on Aging cohort.^[11]^ Data for UC were acquired from the FinnGen research project which includes 6435 cases and 446,419 controls.^[12]^ The diagnosis of UC was made using the Tenth Revision of the International Classification of Diseases (ICD-10) codes. All data for GWAS is sourced from different alliances or agencies, so there is no duplication between samples. The definitions of exposure and outcome are detailed in the original article.

### 2.3. Statistical analysis

To explore the causal relationship between gut microbiota and UC, a bidirectional two-sample MR analysis was conducted. A two-step MR analysis was performed to assess the potential mediation via metabolites in this causal relationship. If no indication of directional pleiotropy was found, the main analysis would utilize the inverse-variance weighted (IVW) method, as it is considered the most reliable when there is no directional pleiotropy.^[13]^ Given the absence of horizontal pleiotropy, the IVW method was applied as the primary means for calculating causal effect estimates to ensure unbiased results. To verify the consistency of the results, complementary methods such as MR-Egger regression, weighted median, simple mode, and weighted mode were also applied.^[13]^ The results were expressed as odds ratios (OR) with 95% confidence intervals (CI) per standard deviation.

Mediation proportions were calculated using the following formula: (β1 × β2)/βall, where βall represents the total causal effects of gut microbiota on UC derived from the main analysis; β1 represents the estimated effect of gut microbiota-related traits on potential plasma metabolites mediators, and β2 represents the causal effect of plasma metabolite mediators on UC.

Cochran Q method was used to assess heterogeneity, with a *P*-value lower than .05 indicating heterogeneity.^[14]^ Cochran Q is a heterogeneity statistic for the IVW model. A Q statistic much larger than its degrees of freedom provides evidence of heterogeneity and potentially invalid IVs.^[15]^ MR-Egger regression was used to identify potential horizontal pleiotropy, and the MR pleiotropy residual sum and outlier (MR-PRESSO) analysis was conducted to minimize possible confounding factors.^[16]^ To investigate the impact of individual SNPs on causal associations, we performed leave-one-out sensitivity tests by removing each SNP 1 at a time.^[17]^ Additionally, scatter plots and funnel plots were generated to assess the robustness of the MR results.

We used MetaboAnalyst 6.0 (https://www.metaboanalyst.ca/) to perform the enrichment analysis of identified metabolites. The model diagram was created using Biorender (https://www.biorender.com/).

### 2.4. IVs selection

The selection of IVs for gut microbiota and plasma metabolites was based on a significance threshold of 1 × 10^-5^, while for UC, the threshold was set at 5 × 10^-8^, in accordance with recent literature.^[18]^ SNPs were clustered to mitigate linkage disequilibrium (window size = 10,000 kb, *R*^2^ < 0.001), ensuring robust and independent genetic instruments for analysis.

## 3. Results

### 3.1. Bidirectional two-sample MR analyses between gut microbiota and UC

**Figure 2.**
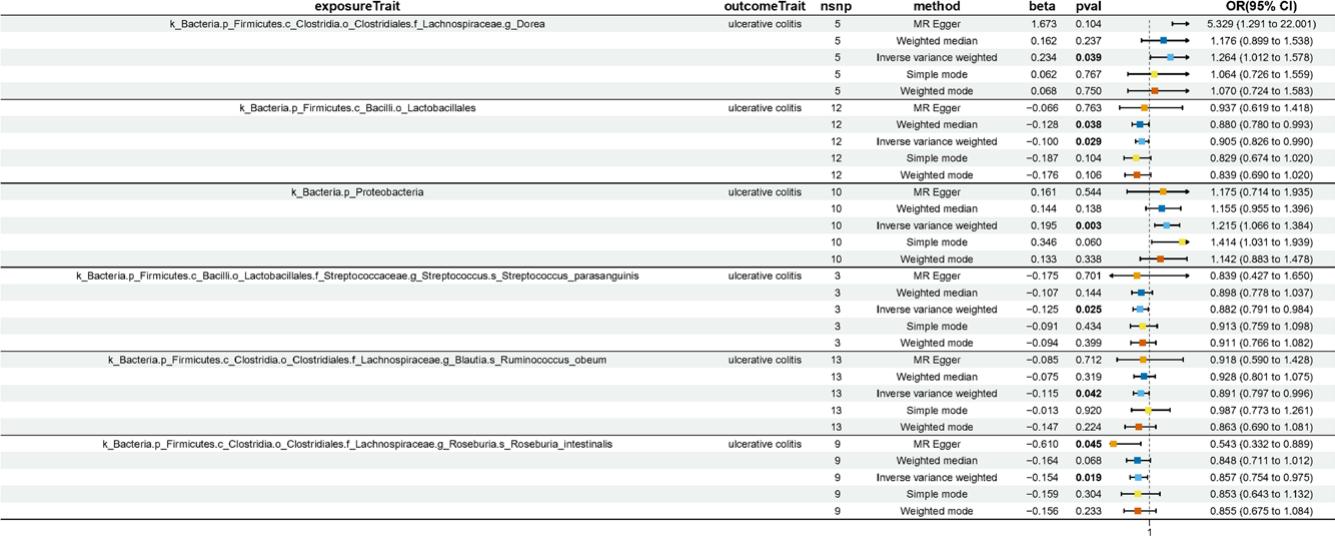
Forest plots of causal relationship of gut microbiota and UC. CI = confidence interval; OR = odds ratio; nSNP = number of single nucleotide polymorphism; *P*val = *P*-values, UC = ulcerative colitis.

Through a two-sample MR analysis, we identified significant associations between specific gut microbial taxa and the risk of UC. In Tables S2, Supplemental Digital Content, https://links.lww.com/MD/P157, 54 SNPs for 6 gut microbiota are presented in detail. Certain microbial taxa exhibited a positive correlation with UC risk, while others showed a protective effect. Genus *Dorea* (OR = 1.264, 95% CI: 1.012–1.578, *P* = .039) and Phylum Proteobacteria (OR = 1.215, 95% CI: 1.066–1.384, *P* = .003) were found to be positively associated with UC risk. In contrast, Species *Streptococcus parasanguinis* (OR = 0.882, 95% CI: 0.791–0.984, *P* = .025), Species *Ruminococcus obeum* (OR = 0.891, 95% CI: 0.797–0.996, *P* = .042), Species *Roseburia intestinalis* (OR = 0.857, 95% CI: 0.754–0.975, *P* = .019), and Order_Lactobacillales (OR = 0.905, 95% CI: 0.826–0.990, *P* = .029) demonstrated inverse associations with UC risk (Fig. 2). The MR results between gut microbiota and UC are described in detail in Tables S2, S3, and S4, Supplemental Digital Content, https://links.lww.com/MD/P157. The reverse MR analysis failed to detect any significant causal effects of the genetic predisposition to UC on the 6 gut microbiota mentioned previously. The *P*-values, which were all >.05 as assessed by the IVW method, suggest that there is no evidence of a reverse causal relationship between the genetic factors for UC and these specific gut microbiota (Tables S5, Supplemental Digital Content, https://links.lww.com/MD/P157). Forest plots and Scatter plots of MR analysis between each gut microbiota and UC are shown in Figures S1 to S4, Supplemental Digital Content, https://links.lww.com/MD/P158.

### 3.2. Two-sample MR analyses between human plasma metabolites and UC

**Figure 3.**
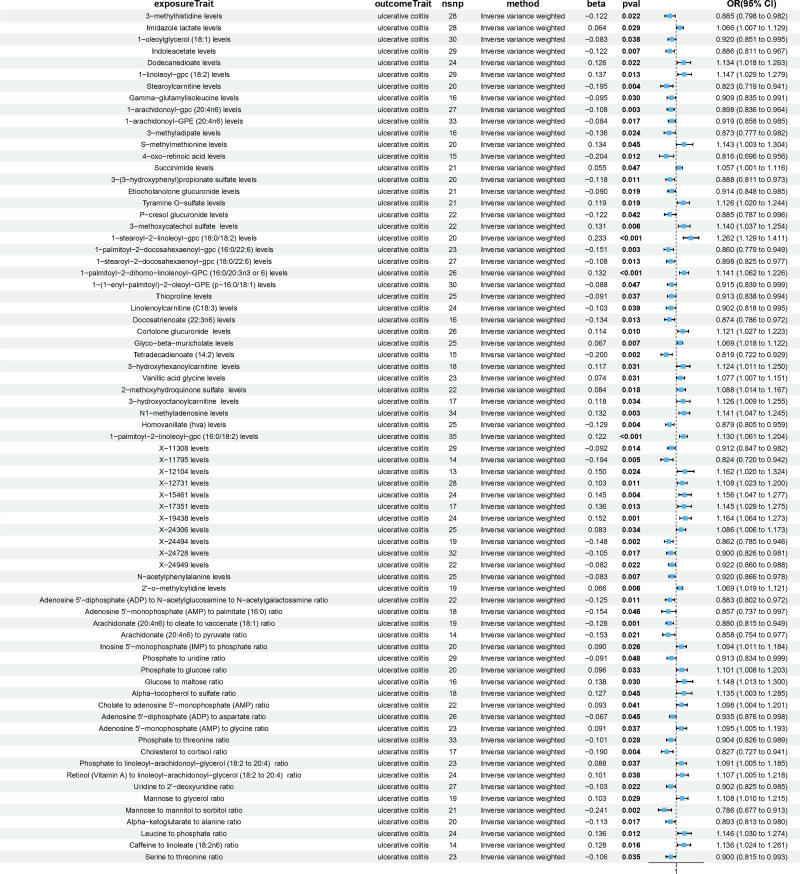
Forest plots of causal relationship of metabolites and metabolite ratios and UC. CI = confidence interval; OR = odds ratio; nSNP = number of single nucleotide polymorphism; *P*val = *P*-values, UC = ulcerative colitis.

**Figure 4.**
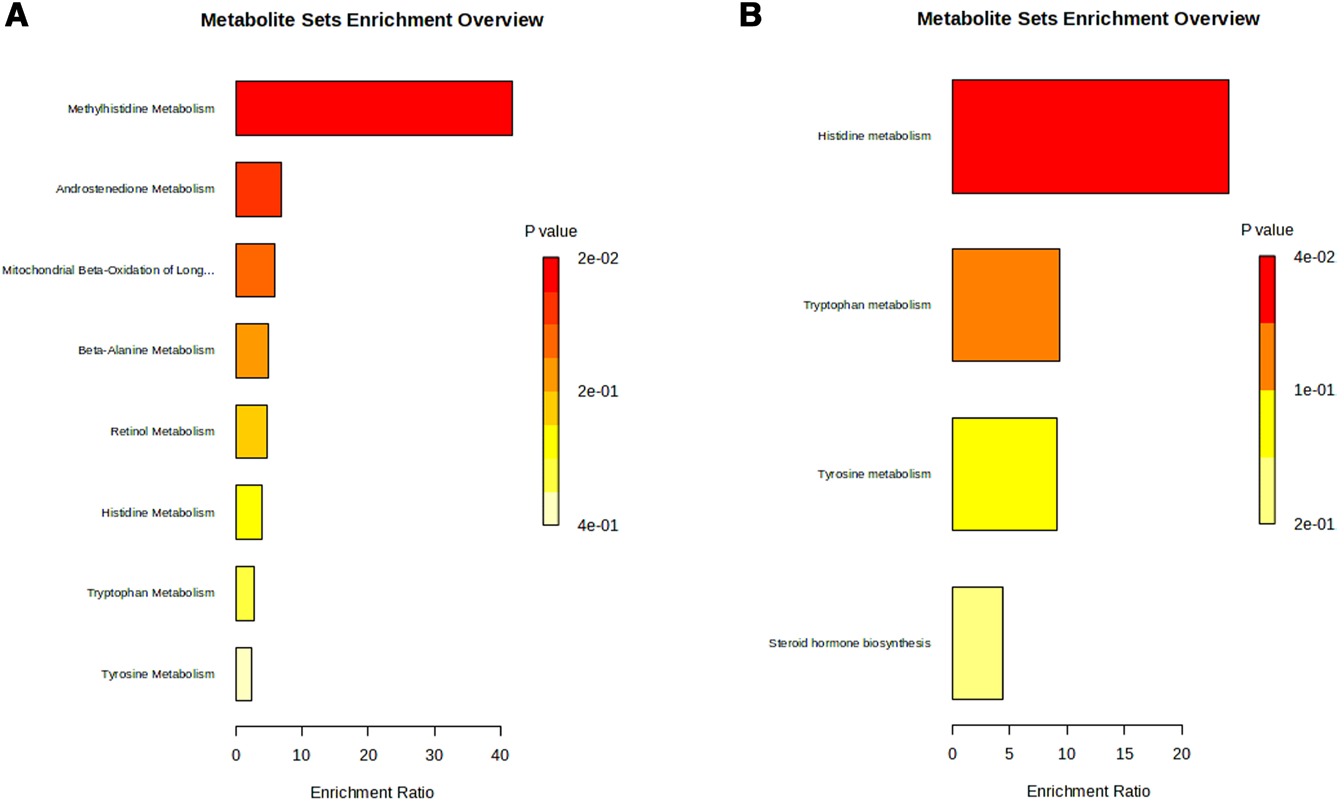
Enrichment analysis results of the causal effect of plasma metabolites on UC. (A) Using Small Molecule Pathway Database (SMPDB); (B) using the Kyoto Encyclopedia of Genes and Genomes (KEGG) database. UC = ulcerative colitis.

Metabolites associated with UC are summarized in Figure 3, in which 73 metabolites and metabolite ratios were identified using the IVW method, including 35 positive and 38 negative causal effects on UC. Among these, there were 39 known metabolites, including 21 lipids and lipid-like molecules, 11 organic acids, 3 organoheterocyclic compounds, 2 nucleosides, 1 organic oxygen compound, 1 benzenoid. Based on known metabolites, enrichment analysis indicated that they were enriched in the Histidine metabolism (Fig. 4A and B). Furthermore, 23 metabolite ratios and 11 unknown metabolites were associated with the UC. Only nominally significant effects were detected rather than statistically significant effects. The MR results between metabolites and UC are described in detail in Tables S6, Supplemental Digital Content, https://links.lww.com/MD/P157.

### 3.3. Causal effects of the selected gut microbiota on the human plasma metabolites

**Figure 5.**
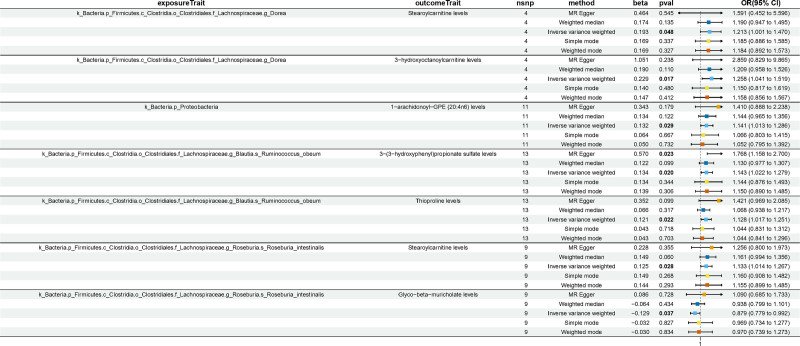
Forest plots of causal relationship of gut microbiota and metabolites and metabolite ratios. CI = confidence interval; OR = odds ratio; nSNP = number of single nucleotide polymorphism; *P*val = *P*-values.

We conducted a two-sample MR analysis on the gut microbiota and metabolites previously screened for association with UC, excluding the proportions of metabolites and unknown metabolites. We included 6 gut microbiota and 39 metabolites for analysis and identified 7 causal relationship combinations between gut microbiota and metabolites (Fig. 5). Genus *Dorea* showed a causal link with elevated levels of Stearoylcarnitine (OR = 1.213, 95% CI: 1.001–1.470, *P* = .048) and an increase in 3-hydroxyoctanoylcarnitine levels (OR = 1.258, 95% CI: 1.041–1.519, *P* = .017). The presence of Phylum Proteobacteria indicated a causal relationship with an increase in 1-arachidonoyl-GPE (20:4n6) levels (OR = 1.141, 95% CI: 1.013–1.286, *P* = .029). Species *R obeum* was found to be causally related to a rise in 3-(3-hydroxyphenyl)propionate sulfate levels (OR = 1.143, 95% CI: 1.022–1.279, *P* = .020) and also to higher thioproline levels (OR = 1.128, 95% CI: 1.017–1.251, *P* = .022). Species *R intestinalis* demonstrated a causal effect on Stearoylcarnitine levels, which were higher (OR = 1.133, 95% CI: 1.014–1.267, *P* = .028), and interestingly, a decrease in Glyco-beta-muricholate levels (OR = 0.879, 95% CI: 0.779–0.992, *P* = .037). The MR results between gut microbiota and metabolites are described in detail in Tables S8, Supplemental Digital Content, https://links.lww.com/MD/P157.

### 3.4. Mediation effects of the selected human plasma metabolites between gut microbiota and UC

**Figure 6.**
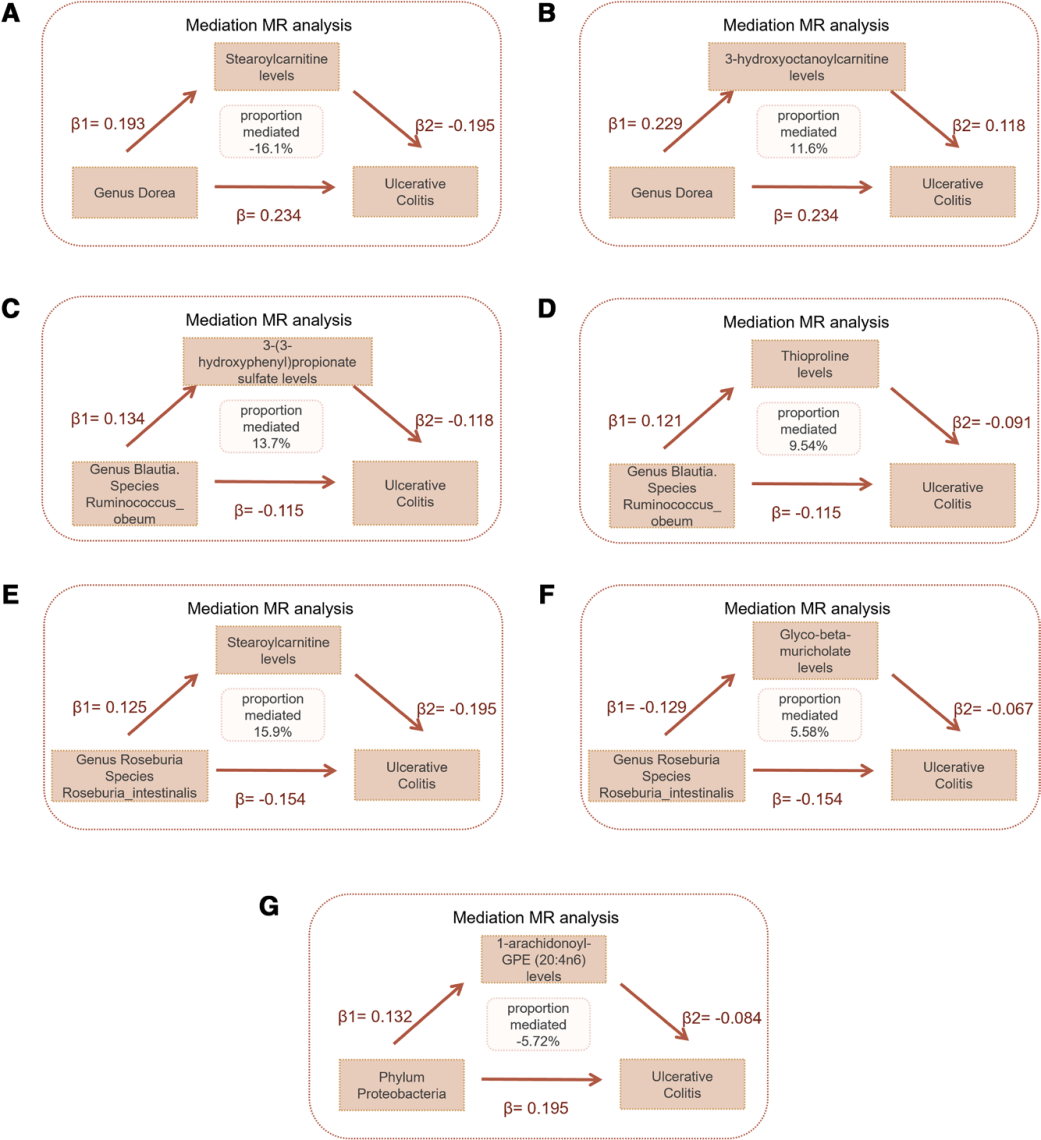
Mediation effects of the selected human plasma metabolites between gut microbiota and UC. (A) Mediation effect of Stearoylcarnitine levels between Genus *Dorea* and UC. (B) Mediation effect of 3-hydroxyoctanoylcarnitine levels between Genus *Dorea* and UC. (C) Mediation effect of 3-(3-hydroxyphenyl)propionate sulfate levels between Species *Ruminococcus obeum* and UC. (D) Mediation effect of Thioproline levels between Species *Ruminococcus obeum* and UC. (E) Mediation effect of Stearoylcarnitine levels between Species *Roseburia intestinalis* and UC. (F) Mediation effect of Glyco-beta-muricholate levels between Species *Roseburia intestinalis* and UC. (G) Mediation effect of 1-arachidonoyl-GPE (20:4n6) levels between Phylum Proteobacteria and UC. UC = ulcerative colitis.

A two-step MR approach was employed, utilizing the identified gut microbial taxa and plasma metabolites, to investigate the potential mediating effects of plasma metabolites in the relationship between gut microbiota and UC. We conducted a mediation analysis to explore the intermediary role of plasma metabolites within these associations. Stearoylcarnitine levels mediated -16.1% of the effect of Genus *Dorea* on UC (Fig. 6A), while 3-hydroxyoctanoylcarnitine levels mediated 11.6% of the same effect (Fig. 6B). 3-(3-hydroxyphenyl)propionate sulfate levels mediated 13.7% (Fig. 6C) and thioproline levels mediated 9.54% (Fig. 6D) of the effect of Species *R obeum* on UC. Stearoylcarnitine levels mediated 15.9% (Fig. 6E) and glyco-beta-muricholate levels mediated 5.58% (Fig. 6F) of the effect of Species *R intestinalis* on UC. 1-arachidonoyl-GPE (20:4n6) levels mediated −5.72% of the effect of Phylum Proteobacteria on UC (Fig. 6G).

### 3.5. Sensitivity analyses

The potential for weak instrument bias in these IVs is minimal, as evidenced by the F-statistics for the chosen SNPs, each exceeding the threshold of 10. There is an absence of linkage disequilibrium, with the SNPs being evenly distributed, as indicated by *R*^2^ values spanning from 0 to 1 (refer to Table S2, Supplemental Digital Content, https://links.lww.com/MD/P157). To evaluate the variability of our estimates, we performed Cochrane Q test, yielding Q statistics and corresponding *P*-values. Our analysis revealed no significant heterogeneity. Sensitivity analyses were conducted to address and correct for possible directional pleiotropy within the causal estimates. The aforementioned MR analyses, which reported non-significant intercepts, suggest no directional pleiotropy. Additionally, a leave-one-out analysis was implemented to identify any substantial deviation by a specific SNP from the overall causal estimate, assessing each SNP’s impact on the aggregate causal effect. After excluding outliers and reducing heterogeneity using the MR-PRESSO global test, our positive findings remained robust. Funnel plots and leave-one-out plots, along with the related data, are all summarized in Figures S5 to S8, Supplemental Digital Content, https://links.lww.com/MD/P158, and Tables S4, S7, and S9, Supplemental Digital Content, https://links.lww.com/MD/P157.

## 4. Discussion

In this study, we systemically investigated the causal effects of the gut microbiota and plasma metabolites on UC. We revealed that 6 taxa and 73 metabolites and metabolite ratios were causally associated with UC. Further analysis showed that 4 taxa affected UC via 6 mediation metabolites, which could serve as potential biomarkers for risk stratification and as underlying mechanisms for further investigation of UC.

Through bidirectional two-sample MR analyses, we identified 4 taxa, including Genus *Dorea*, Genus *Blautia* Species *R obeum*, Genus *Roseburia* Species *R intestinalis*, Phylum Proteobacteria that have a causal relationship with UC. Among them, the first 3 genera all belong to the Family Lachnospiraceae.

There is controversy in previous articles regarding the relative levels of Lachnospiraceae in patients with UC compared to healthy individuals.^[19,20]^ In patients with IBD, including Crohn disease and UC, the abundance of some strains of Lachnospiraceae species increased, while the abundance of other species decreased.^[21]^ This suggests that specific members of Lachnospiraceae may be associated with the development of IBD. The Lachnospiraceae accounts for approximately 10% of the total gut microbiota. In terms of metabolic contributions and immune modulation, the bacterial family Lachnospiraceae is notable for its production of SCFAs. Butyrate, a key SCFA, is predominantly produced by the *Roseburia*, particularly under slightly acidic conditions, and is accompanied by the utilization of acetate.^[22]^ These SCFAs are pivotal in governing the carbon metabolism of intestinal epithelial cells and in the stimulation of regulatory T cells, which are essential for maintaining gut homeostasis and immune tolerance.^[23]^ Sasaki et al^[24]^ detected that the relative abundance of Lachnospiraceae is not correlated with the disease activity of UC patients (such as the partial Mayo score). This suggests that although the quantity of Lachnospiraceae is reduced in UC patients, this reduction is not related to changes in disease activity. Vacca et al^[25]^determined that the abundance of Lachnospiraceae in the gut microbiome was not altered by UC. Based on their findings, they suggested that the diminished presence of Lachnospiraceae and the associated decline in butyrate synthesis might be instrumental in the onset of UC relapses. The study of Wu et al showed bacteria from the Lachnospiraceae family are associated with the success of FMT treatment, particularly playing a positive role in microbial communities linked to the remission of UC.^[26]^

Genus *Dorea* improves UC conditions by managing the concentrations of plasma stearoylcarnitine and 3-hydroxyoctanoylcarnitine. Stearoylcarnitine and 3-hydroxyoctanoylcarnitine are both acylcarnitine compounds that play a pivotal role in the metabolism of fatty acids, particularly in their transport and oxidation processes. These acylcarnitines are integral to cellular energy metabolism, as they facilitate the translocation of long-chain fatty acids into the mitochondria for β-oxidation, thereby providing energy for cellular activities.^[27]^ The accumulation of long-chain acylcarnitines may indicate disruptions in the fatty acid oxidation process, potentially due to mitochondrial dysfunction caused by inflammation. Changes in acylcarnitine levels could serve as biomarkers for the activity of UC, aiding in the monitoring of disease status and treatment responses. This may contribute to the development of new diagnostic tools and therapeutic strategies.^[28]^

Species *R obeum* within the *Blautia* genus negatively impacts UC by altering plasma levels of 3-(3-hydroxyphenyl)propionate sulfate and thioproline. *Blautia* has demonstrated potential probiotic properties, capable of alleviating colitis induced by dextran sulfate sodium in mice through several mechanisms. These include the suppression of inflammatory responses, maintenance of the intestinal barrier, inhibition of the TLR4/NF-κB signaling pathway, and regulation of the balance within the gut microbiota. This bacterium’s multifaceted approach to mitigating colitis highlights its promise as a probiotic candidate for managing intestinal inflammation.^[29]^ However, some studies present findings that contrast with our research outcomes. Certain research has indicated that *Blautia* may exacerbate colitis in mouse models, suggesting that under specific circumstances, particular species of *Blautia* could be associated with the pathogenesis of IBD. This highlights the complexity of microbial influences in UC and the potential for species within the same genus to have divergent effects on intestinal inflammation.^[30]^ 3-(3-hydroxyphenyl)propionate sulfate, known as 3-HPPA sulfate, is a microbial metabolite of quercetin, a flavonoid abundant in plants. Poorly absorbed in the upper gastrointestinal tract when ingested, quercetin is converted by the gut microbiota into various phenolic acids, with 3-HPPA being 1 of them. Research indicates that 3-HPPA mitigates the adhesion of monocytes to endothelial cells by regulating the expression of E-selectin, a crucial initial step in atherosclerosis development. Additionally, 3-HPPA has been shown to suppress the upregulation of E-selectin and other cellular adhesion molecules induced by tumor necrosis factor α, and it inhibits the activation of the NF-κB signaling pathway, thus reducing inflammatory responses. By suppressing the NF-κB pathway, 3-HPPA provides additional evidence for the health benefits of dietary flavonoids and their microbial metabolites as potential therapeutic agents for atherosclerosis.^[31]^ Thioproline, a sulfur-containing amino acid derived from the reaction between cysteine and formaldehyde, offers a variety of potential health benefits within the human body. Research indicates that thioproline acts as a potent antioxidant, capable of safeguarding human cells against oxidative stress and enhancing cellular vitality. Moreover, its effective nitrite-trapping ability suggests that thioproline may play a significant role in cancer prevention. This amino acid, found in various cooked foods such as vegetables, cod, shiitake mushrooms, and liver-based dishes, particularly in high concentrations in djenkol beans and certain leguminous beans, could have a positive impact on health by its presence in the diet. Thioproline, a sulfur-containing amino acid derived from the reaction between cysteine and formaldehyde, offers a variety of potential health benefits within the human body. Research indicates that thioproline acts as a potent antioxidant, capable of safeguarding human cells against oxidative stress and enhancing cellular vitality.^[32]^ Moreover, this strong nitrite-trapping property also makes thioproline a potentially active agent against the development of cancer actively.^[33]^ Our research revealed that *R obeum* from the genus *Blautia* enhances UC by increasing 3-(3-hydroxyphenyl)propionate sulfate and thioproline. It may represent a potential mechanism to be carried out in further studies.

The genus *Roseburia*, specifically *R intestinalis*, exacerbates UC symptoms by modulating plasma stearoylcarnitine and glyco-beta-muricholate. *Roseburia* is one of the most important genera in gut microbiota and thus almost completely is linked with human health and disease states. In UC patients, its abundance is usually cut down and is associated with a decrease in SCFAs production. SCFAs, and among them butyrate, are essential for intestinal barrier functions, immune response modulation, and suppression of inflammatory processes. Indeed, several studies have demonstrated that *Roseburia* produces SCFAs-a remarkably high amount of butyrate-which serves as a nutritional molecule for colonic epithelial cells. This carboxylic acid decreases the colonic pH, thus stimulating epithelial cell proliferation and inhibiting growth of cancerous cells, enhancing mucin, antimicrobial peptides, and tight junction protein production, hence enhancing intestinal barrier properties.^[34]^ *Roseburia* is also known to induce anti-inflammatory regulatory T cells that may therefore play an important role, especially in the GI tract, in regulating inflammatory processes.^[35]^ The importance of *Roseburia* both for maintaining intestinal health and in the development and treatment of UC brings attention to the possibility of manipulating the gut microbiota as a means to manage these conditions. Glyco-beta-muricholate represents a conjugated bile acid-major constituents of bile, derived from cholesterol metabolism by the liver. These are biologically active steroid molecules. Glyco-beta-muricholate plays an important role in enterohepatic recirculation of bile acid. In the small intestine, it promotes the digestion and absorption of lipids. In the colon, primary conjugated bile acids could be converted to secondary bile acids by microbial enzymes. It is important for the balance between gut microbiota and regulation of host metabolism and inflammation responses.^[36]^ Also, glyco-beta-muricholate is a conjugate of β-muricholate with taurine. Both these bile acids might be able to modulate gut microbiota, preserving intestinal barrier integrity. Nevertheless, deeper research into their exact physiological roles and mechanism of action will be required for complete comprehension.

Additionally, our research found that the phylum Proteobacteria is also causally related to UC in a positive correlation. Proteobacteria is the largest phylum of bacteria, including many pathogenic species, such as *Escherichia coli*, *Salmonella*, *Vibrio cholerae*, *Helicobacter pylori*, and other well-known types, the vast majority of which are pathogenic. All Proteobacteria are gram-negative bacteria, and their outer membrane is mainly composed of lipopolysaccharides.^[37]^ In the gut microbiota of patients with UC, the relative abundance of Proteobacteria is usually high. Some bacteria within the Proteobacteria may play a promoting role in UC. *E coli*, a common pathogen in the Proteobacteria phylum, has been found in studies to potentially affect intestinal epithelial cells in UC by producing cytotoxins, such as Cytolethal Distending Toxin. This can lead to cell cycle arrest, activation of DNA damage response, and ultimately may cause cell death or senescence, increasing the risk of tumorigenesis.^[38]^ Additionally, the Proteobacteria phylum may also have an impact on the psychology of patients with IBD. Humbel et al^[39]^found that in IBD patients, psychological distress is associated with a reduced abundance of Betaproteobacteria and Gammaproteobacteria in the gut, which may reflect the interaction of the gut-brain axis. Phylum Proteobacteria exerts a positive influence on UC by regulating levels of 1-arachidonoyl-GPE (20:4n6). 1-arachidonoyl-GPE (1-arachidonoyl-sn-glycero-3-phosphoethanolamine) is a type of phospholipid, belonging to the class of glycerophospholipids. Research has indicated that its levels are positively correlated with the preservation of residual β-cell function in patients with type 1 diabetes following FMT. Additionally, its levels show a positive correlation with changes in the bacteria *Desulfovibrio piger* in the small intestine, suggesting that it may exert its effects by influencing specific gut microbes. The probable mechanism involves modulating the function of particular immune cell subsets, such as CD4^+^ CXCR3^+^ T cells, thereby impacting the progression of the disease.^[40]^ Our research suggests that it plays a mediating role in the impact of the Proteobacteria phylum on UC, indicating a potential future research prospect for its involvement in the initiation and development of inflammation.

This study has several advantages. Initially, we harnessed aggregated data from the GWAS consortium for our MR analyses, thereby amplifying statistical strength as genetic disparities remain impervious to the influence of confounding elements. Additionally, we integrated bidirectional and sequential MR methodologies to scrutinize the possible mediating roles of metabolites, yielding outcomes of enhanced precision and dependability. This pioneering strategy offers an exhaustive insight into the causal nexus between genetic markers and UC.

When interpreting the findings of our study, several limitations must be taken into account. Firstly, we employed a less stringent threshold of 1 × 10^-5^ to identify instrument variables for certain exposures. Nonetheless, the F-statistics in our analysis surpassed the benchmark of 10, suggesting that any potential bias from weak instruments was likely to be minimal. Secondly, our study was confined to populations of European ancestry, which implies that the applicability of our results to non-European populations should be approached with caution. The genetic correlations between gut microbiota and host genomes may exhibit variations across different ethnicities, and the sample size of the GWAS summary data might not have been large enough to fully elucidate all possible causal relationships. Despite these considerations, our findings, which identified independent genetic variants associated with gut microbiota traits at a genome-wide significance level, provide valuable insights into the complex interactions within the gut microbiome.

## 5. Conclusion

Our MR study concludes that we have identified 6 gut microbiota that may have a causal relationship with UC, and 4 taxa among them have significant genetic correlations mediated by plasma metabolites. These findings provide a new perspective on the research into the etiology and treatment of UC, involving the gut microbiome and metabolites, and propose potential intervention targets for UC. To validate these results and understand the underlying mechanisms, further investigative research is required.

## Acknowledgments

We thank all the teams that contributed to the GWAS data involved in this study.

## Author contributions

**Conceptualization:** Shidong Zhao, Xianjing Zheng, Changjiang Yang, Weisong Shen, Zhanlong Shen.

**Data curation:** Shidong Zhao, Xianjing Zheng, Zhanlong Shen.

**Formal analysis:** Shidong Zhao, Xianjing Zheng, Changjiang Yang, Zhanlong Shen.

**Funding acquisition:** Zhanlong Shen.

**Investigation:** Shidong Zhao, Xianjing Zheng, Changjiang Yang, Zhanlong Shen.

**Methodology:** Shidong Zhao, Xianjing Zheng, Changjiang Yang, Zhanlong Shen.

**Supervision:** Weisong Shen.

**Validation:** Xianjing Zheng, Changjiang Yang, Weisong Shen.

**Visualization:** Xianjing Zheng, Changjiang Yang, Weisong Shen.

**Writing – original draft:** Shidong Zhao, Xianjing Zheng, Zhanlong Shen.

**Writing – review & editing:** Shidong Zhao, Xianjing Zheng, Weisong Shen, Zhanlong Shen.

## Supplementary Material


