# Supplementary material for: Gut microbiota causally affects ulcerative colitis by potential mediation of plasma metabolites: A Mendelian randomization study
Source: Medicine (Baltimore). 2025 Jun 27;104(26):e42791. doi: 10.1097/MD.0000000000042791 (PMC12212764; doi:10.1097/MD.0000000000042791)
Supplement: Supplementary file 2 [file medi-104-e42791-s002.docx]

**
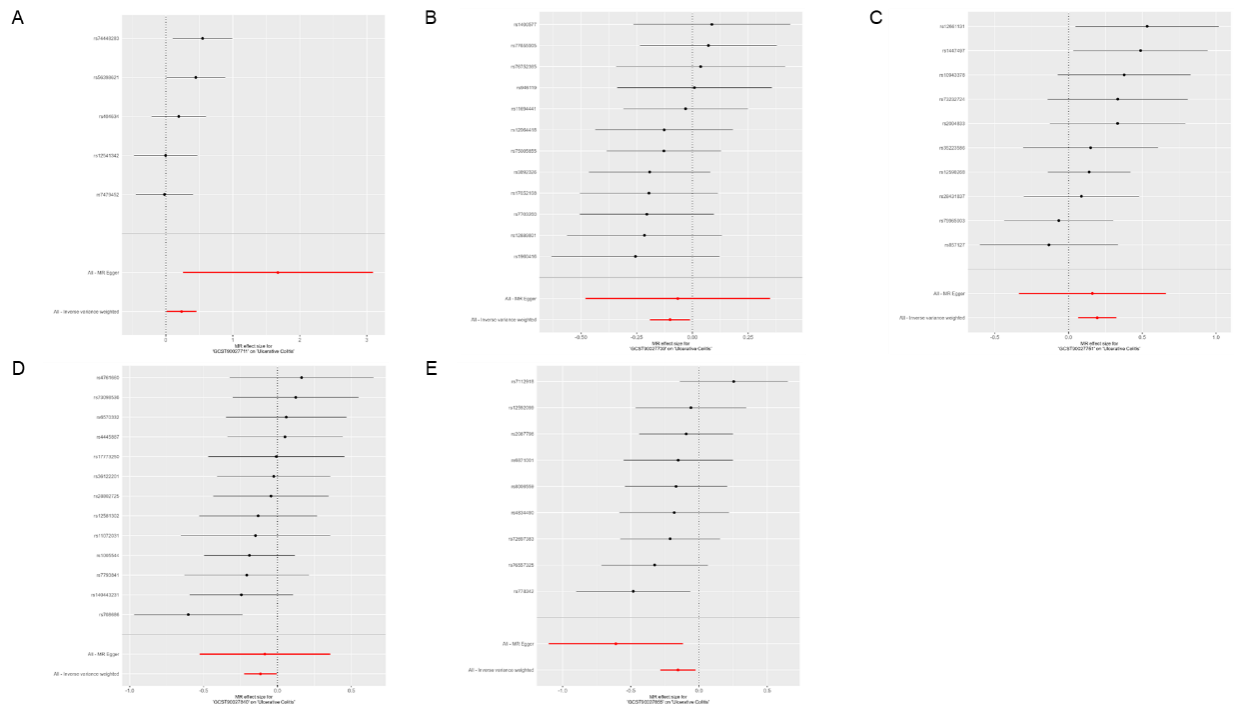
Supplementary Figures (S1~S8)**

**Figure S1.** Forest plots of MR analysis of gut microbiota on UC. (A)Effect of Genus *Dorea* on UC**.** (B)Effect of Order Lactobacillales on UC. (C)Effect of Phylum Proteobacteria on UC. (D)Effect of Species *Ruminococcus obeum* on UC. (E)Effect of Species *Roseburia intestinalis* on UC. There were insufficient instrumental variables for the Species *Streptococcus parasanguinis* to generate a meaningful plot.


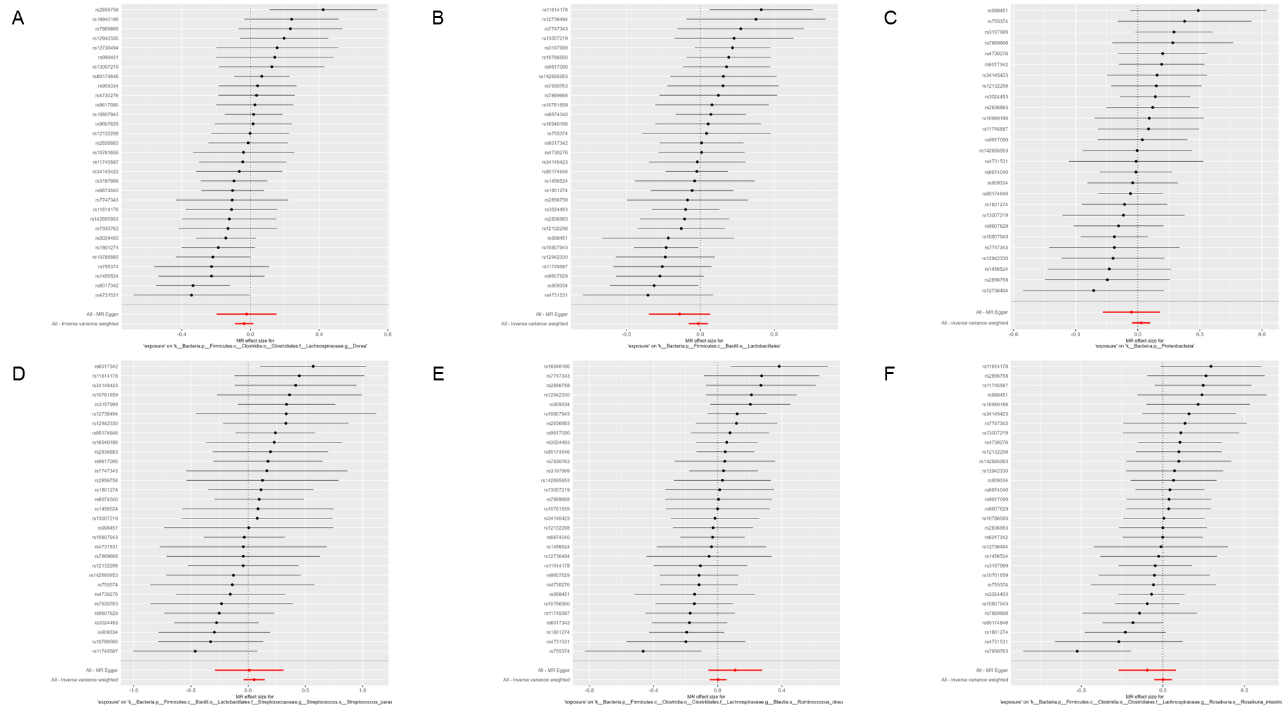
**Figure S2.** Forest plots of MR analysis of UC on gut microbiota.

(A)Effect of UC on Genus *Dorea***.** (B) Effect of UC on Order Lactobacillales. (C) Effect of UC on Phylum Proteobacteria. (D) Effect of UC on Species *Streptococcus_parasanguinis*. (E) Effect of UC on Species *Ruminococcus obeum***.** (F) Effect of UC on Species *Roseburia intestinalis*


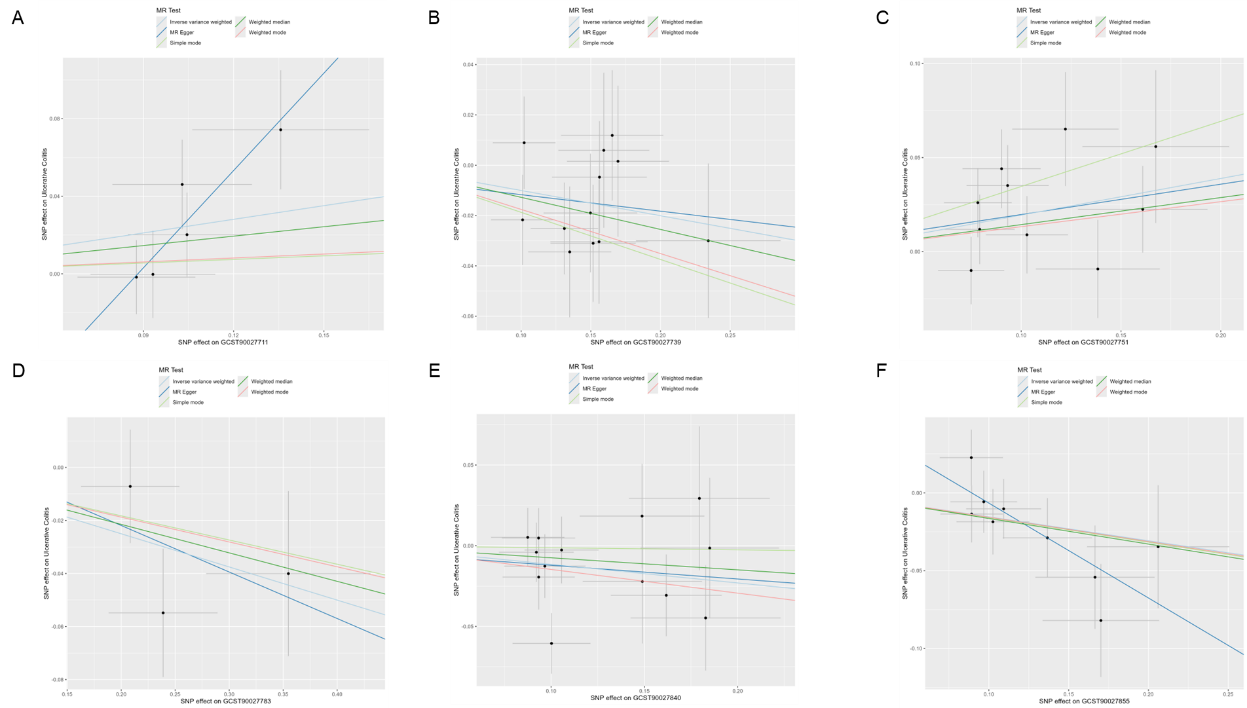


**Figure S3.** Scatter plots of MR analysis of gut microbiota on UC. (A)Effect of Genus *Dorea* on UC**.** (B)Effect of Order Lactobacillales on UC. (C)Effect of Phylum Proteobacteria on UC. (D) Effect of Species *Streptococcus_parasanguinis* on UC. (E)Effect of Species *Ruminococcus obeum* on UC. (F)Effect of Species *Roseburia intestinalis* on UC.


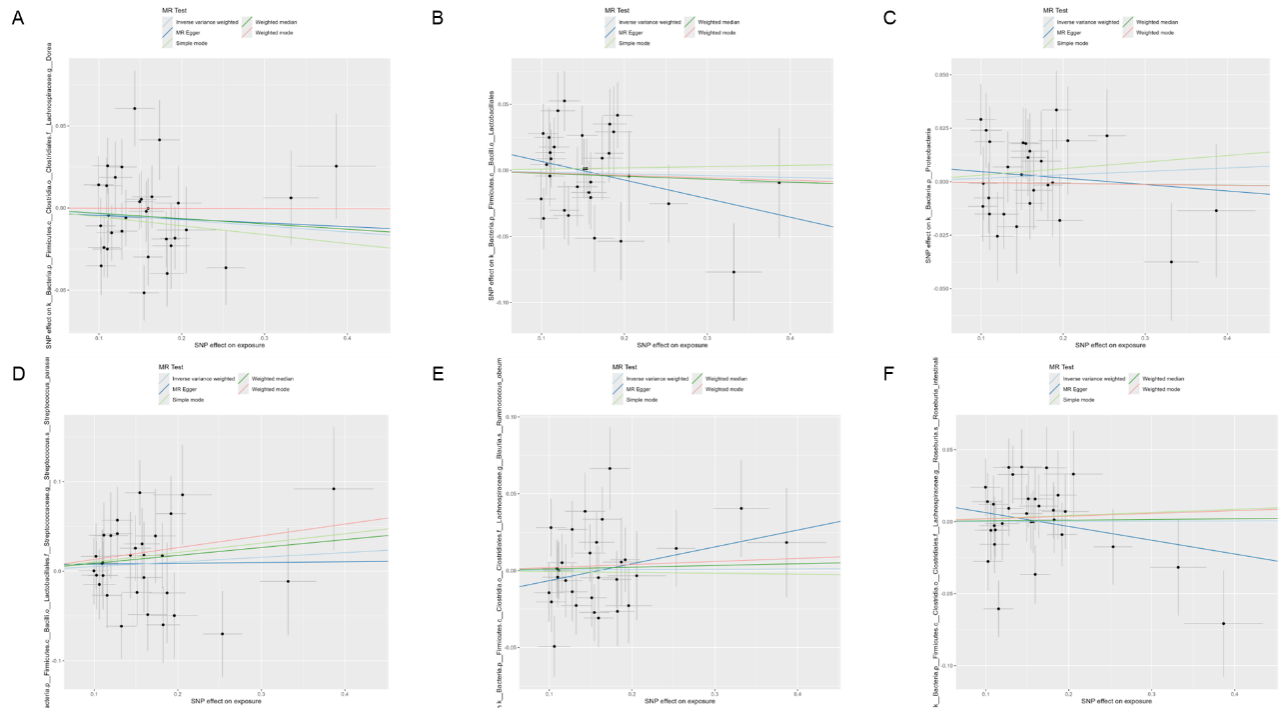


**Figure S4.** Scatter plots of MR analysis of UC on gut microbiota.

(A)Effect of UC on Genus *Dorea***.** (B) Effect of UC on Order Lactobacillales. (C) Effect of UC on Phylum Proteobacteria. (D) Effect of UC on Species *Streptococcus_parasanguinis*. (E) Effect of UC on Species *Ruminococcus obeum***.** (F) Effect of UC on Species *Roseburia intestinalis*

*
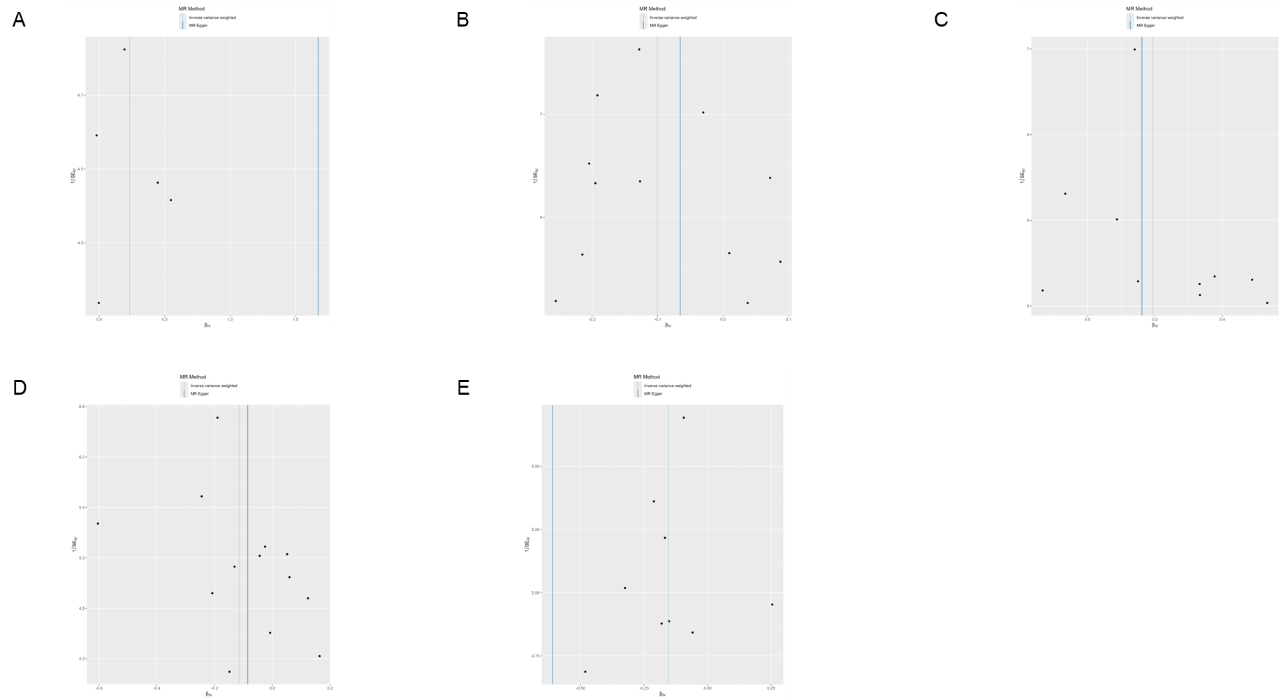
***Figure S5.** Funnel plots of MR analysis of gut microbiota on UC. (A)Effect of Genus *Dorea* on UC**.** (B)Effect of Order Lactobacillales on UC. (C)Effect of Phylum Proteobacteria on UC. (D)Effect of Species *Ruminococcus obeum* on UC. (E)Effect of Species *Roseburia intestinalis* on UC. There were insufficient instrumental variables for the Species *Streptococcus parasanguinis* to generate a meaningful plot.


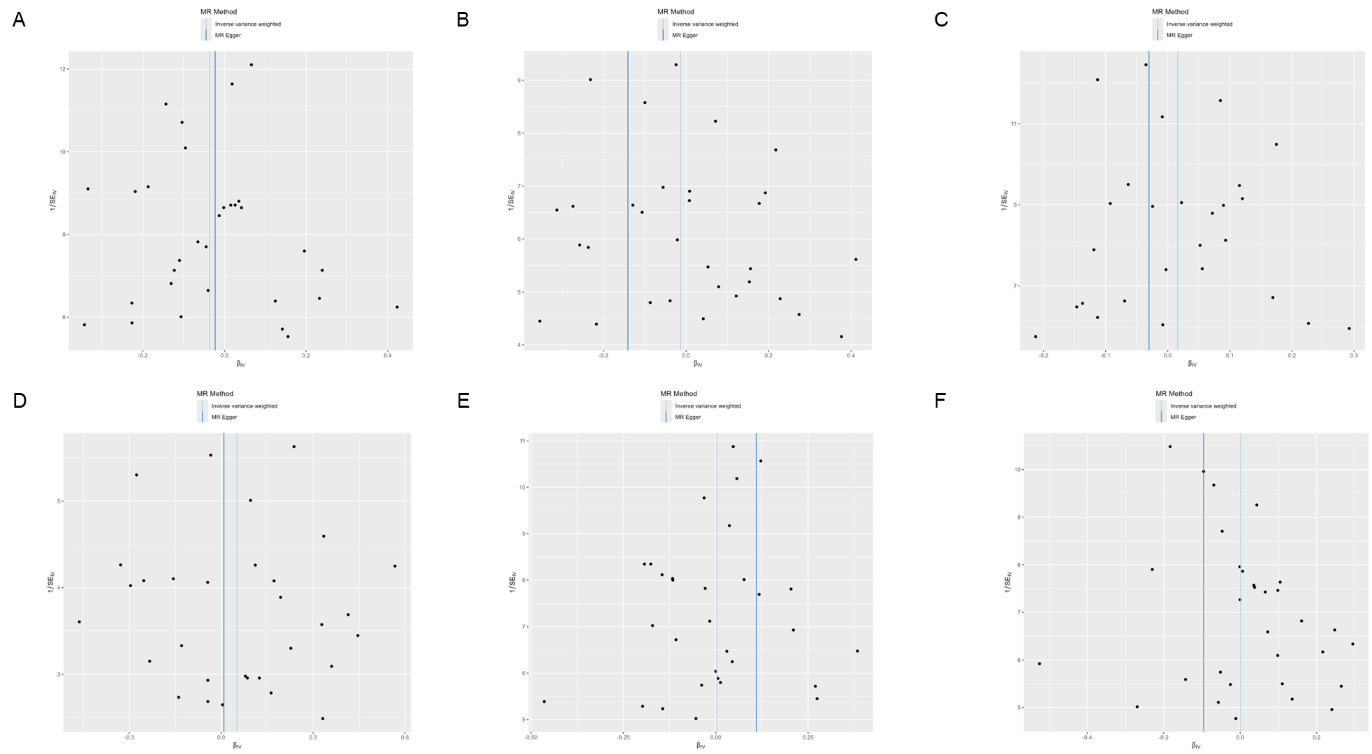


**Figure S6.** Funnel plots of MR analysis of UC on gut microbiota.

(A)Effect of UC on Genus *Dorea***.** (B) Effect of UC on Order Lactobacillales. (C) Effect of UC on Phylum Proteobacteria. (D) Effect of UC on Species *Streptococcus_parasanguinis*. (E) Effect of UC on Species *Ruminococcus obeum***.** (F) Effect of UC on Species *Roseburia intestinalis*


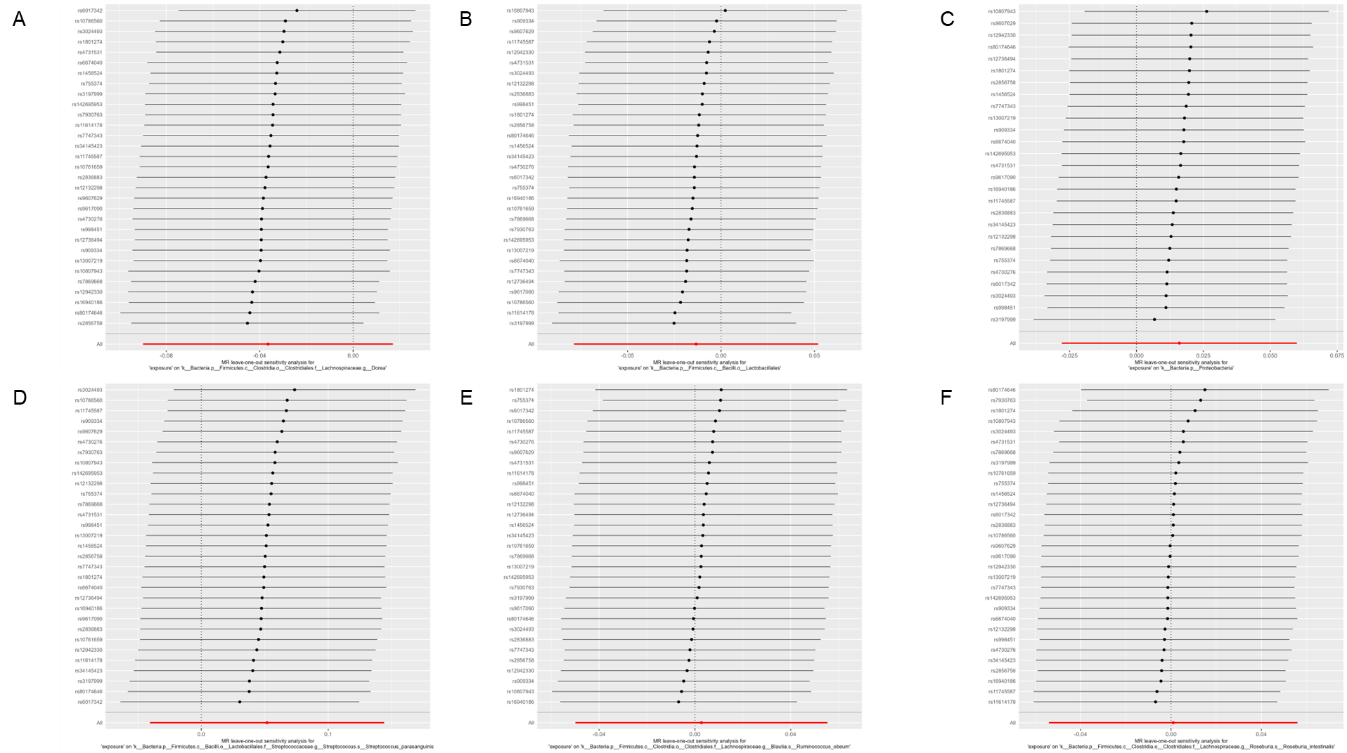

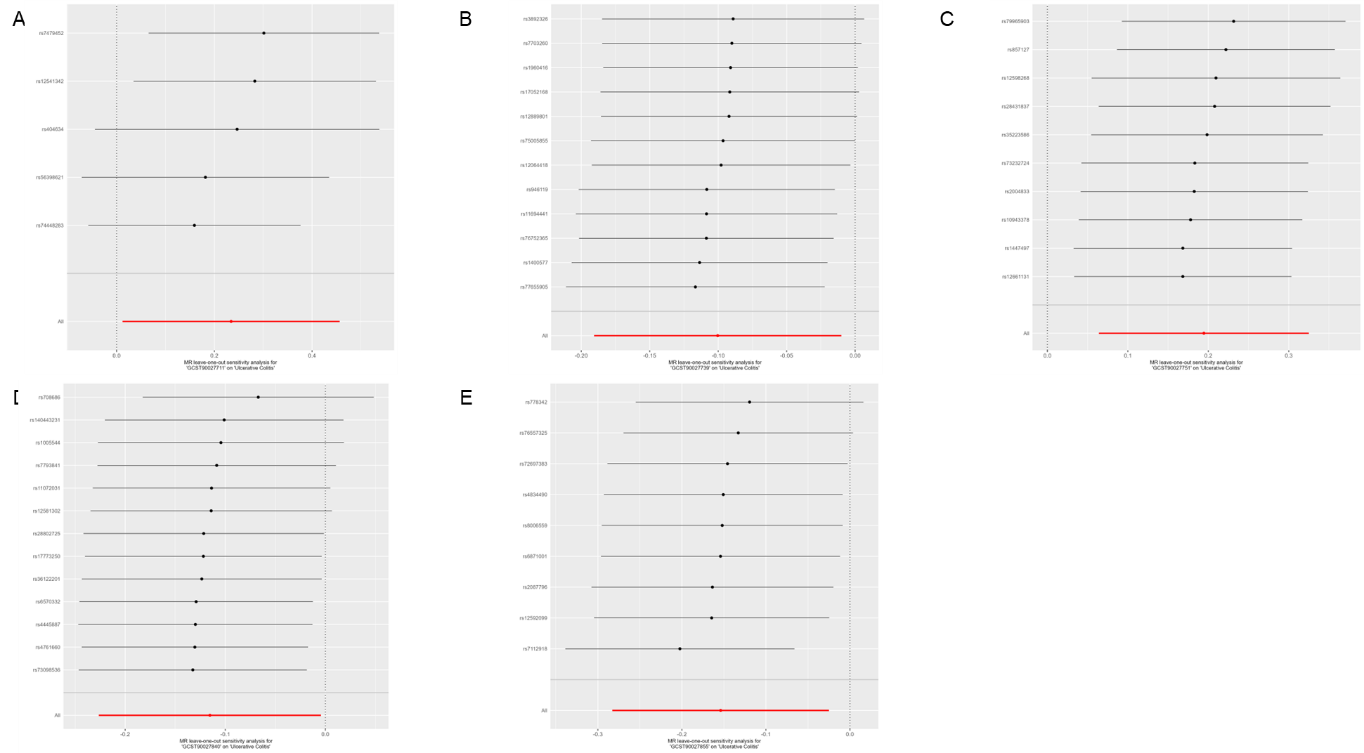
**Figure S7.** Leave-one-out plots of MR analysis of gut microbiota on UC. (A)Effect of Genus *Dorea* on UC**.** (B)Effect of Order Lactobacillales on UC. (C)Effect of Phylum Proteobacteria on UC. (D)Effect of Species *Ruminococcus obeum* on UC. (E)Effect of Species *Roseburia intestinalis* on UC. There were insufficient instrumental variables for the Species *Streptococcus parasanguinis* to generate a meaningful plot.

**Figure S8.** Leave-one-out plots of MR analysis of UC on gut microbiota.

(A)Effect of UC on Genus *Dorea***.** (B) Effect of UC on Order Lactobacillales. (C) Effect of UC on Phylum Proteobacteria. (D) Effect of UC on Species *Streptococcus_parasanguinis*. (E) Effect of UC on Species *Ruminococcus obeum***.** (F) Effect of UC on Species *Roseburia intestinalis*
